# Contributions of the radonorm project to European and international radiation protection research

**DOI:** 10.1007/s00411-025-01156-w

**Published:** 2025-10-18

**Authors:** Warren John, Mandy Birschwilks, Laureline Février, Balázs Madas, Jonne Naarala, Valtteri Nieminen, Aleš Froňka, Tanja Perko, Andrzej Wojcik, Nadja Železnik

**Affiliations:** 1 Federal Office for Radiation Protection, BfS, Ingolstaedter Landstrasse 1 , 85764 Oberschleissheim, Germany; 2 Autorité de Sûreté Nucléaire et de Radioprotection (ASNR), PSE-ENV/SPDR/LT2S, Saint-Paul-lez-Durance, F-13115 France; 3 HUN-REN Center for Energy Research, Konkoly-Thege Miklós út 29-33, 1121 Budapest, Hungary; 4 Department of Environmental and Biological Sciences, University of Eastern Finland, Yliopistonranta 8, Kuopio, 70210 Finland; 5 National Radiation Protection Institute, SURO, Bartoškova 1450/28 4-Nusle, 140 00 Praha, Czech Republic; 6 Belgian Nuclear Research Center, SCK CEN, Boeretang 200, Mol, 2400 Belgium; 7 Stockholm University, Frescativägen, Stockholm, 114 19 Sweden; 8 Elektroinštitut Milan Vidmar, Hajdrihova ulica 2, 1000 Ljubljana, Slovenia

**Keywords:** RadoNorm, Radon, Naturally occurring radionuclides, Radiation protection, EURATOM, Radiation research

## Abstract

RadoNorm has come a long way since its inception, in addressing and managing risks for radon and NORM. The output of the project has been substantial in generating new findings and developing novel methodologies and tools. At the same time, the project has been translating its results into recommendations, which have been published as deliverables and scientific publications, and communicated to various stakeholders through its annual meetings, monthly webinars and the most recent RadoNorm Showcase Meeting held in Brussels. This review aims to produce a comprehensive summary of RadoNorm results and recommendations until this point (June 2025) as well as the recent discussions at the RadoNorm Showcase Meeting in March 2025, where the results were presented to a variety of stakeholders including the radiation research platforms. The results are presented under the themes of “health effects and risks”, “exposure and mitigation” and “risk communication and societal aspects”. RadoNorm has performed a comprehensive assessment of what has been achieved and what future questions have arisen from the latest results of the project, which are also elaborated upon herein and addressed as challenges for the European radiation research platforms to assimilate into their research portfolios.

**Supplementary Information:**

The online version contains supplementary material available at 10.1007/s00411-025-01156-w.

## Introduction to radonorm

The RadoNorm project was conceived to provide new knowledge to effectively manage risks arising from exposure to radon and naturally occurring radioactive material (NORM). This research project, funded through the EURATOM Horizon2020 programme to a total of 18 million euros, brings together 57 reputed European institutions under the coordination of the Federal Office for Radiation Protection, to perform multidisciplinary research in its five scientific work packages to (1) characterise the exposure of humans and non-human biota, (2) provide doses and dose distributions for epidemiological studies, for specific subpopulations and their uncertainties of exposure, (3) increase knowledge of biological effects and their implications on risk assessment, (4) evaluate effectiveness of current mitigation strategies and develop new ones, and (5) incorporate the societal component of developing theory- and evidence-based risk communication, thereby contributing to an improved governance of radon and NORM risks (Kulka et al. 2022). Encompassing this research are also initiatives to provide education and training activities and raise a new generation of researchers, which is essential in the light of declining competence in radiation protection (Rühm et al. 2023). RadoNorm also strives to ensure that its results are effectively communicated across stakeholder groups for their implementation in society and the system of radiation protection.

The results of RadoNorm are mainly relevant to the implementation of the Council Directive 2013/59/EURATOM laying down the European Basic Safety Standards (BSS). The results would shed light on improving and implementing the radon action plans in European Union Member States (EU-MS) by helping to address uncertainties in radon and thoron (Rn-220) metrology, accurately identify radon risk/radon priority areas, assess the impact of the recommended reference levels in a health context and assist authorities to go about communicating radon risks and advising the public to test and mitigate against radon (Martell et al. 2023). The management of NORM risks is still in its infancy compared to the management of radon risks and progress in RadoNorm would help the EU-MS to assess the risks from industries that handle NORM, establish safe clearance levels for NORM-involving processes and assess risks from new sources of NORM, especially in the context of circular economy and the rising NORM-containing products on the market, such as in construction.

RadoNorm efforts contribute significantly to a mechanistic understanding of lung cancer following radiation exposure and assessing the risk to more vulnerable populations, such as children, who are regularly exposed to ionising radiation. Such results would contribute to the report from the United Nations Scientific Committee on the Effects of Atomic Radiation (UNSCEAR 2019) on the effects and risks of ionising radiation, help in developing a new radon handbook for the World Health Organization (WHO) and, more specifically, help the International Agency for Research on Cancer (IARC) in its goals of early cancer detection and cancer prevention in private and occupational contexts (IARC 2001). Moreover, the results would address multiple priorities of the International Atomic Energy Agency (IAEA).

More importantly, RadoNorm has primarily orientated its research foci to the strategic research agendas (SRAs) of four European radiation research platforms: the Multidisciplinary European Low Dose Initiative (MELODI); the European Radioecological ALLIANCE; the European Radiation Dosimetry Group (EURADOS) and the Social Sciences and Humanities in Ionising Radiation Research (SHARE) platform. Whilst the research in the project was conceptualised with the available SRAs prior to 2020 and the joint roadmap from the European Joint Programme CONCERT (Impens and Salomaa 2019), the more recently published SRAs of the platforms (Bouffler et al. 2022; Harrison et al. 2021; Gilbin et al. 2021; SHARE 2020) still include research priorities that are currently addressed by RadoNorm tasks. The multidisciplinary nature and all-roundedness of RadoNorm ensure that all topics are either comprehensively covered (such as in the case of the MELODI SRA), or that a large proportion of the visions and research lines are tackled. The contributions of RadoNorm to each of the SRAs are elaborated on in Overview of achievements and recommendations and contributions to European Radiation Research Platforms. This multidisciplinarity has also managed to break through the boundaries in research fields, enabling a level of collaboration between disciplines to address pressing issues in a way that has never been seen before in radon and NORM research. Additionally, the results may be crucial in promoting stakeholder participation in decision-making processes, updating regulatory frameworks and bolstering their implementation, and enhancing the radiation protection culture across industries, respectively.

The document aims to underline that progress made within RadoNorm has led to measurable contributions to the research priorities of the European radiation research platforms. Whilst the project has been ascertaining and evaluating risks of ionising radiation at low levels of exposure in humans and the environment, new questions have arisen, which require further research for effective radiation protection and implementation into policy. These identified research questions by the RadoNorm consortium have been formulated as challenges for future research, as outlined in Challenges for radon and NORM research, to be incorporated into the SRAs. Moreover, these achievements and challenges have been discussed at length with the wider community during the course of the project and Discussion with the radiation research platforms and other stakeholders will elaborate on the views from other stakeholders.

## Overview of achievements and recommendations and contributions to European radiation research platforms

Out of the 85 scheduled deliverables, RadoNorm results have been published in 58 deliverables and 68 scientific publications to date (June 2025). These results have been presented in a variety of scientific conferences and meetings. This section outlines the main achievements in the project, which are discussed more in detail in the linked publications, and show their contributions to the SRAs of the radiation research platforms.

### Health effects and risks

To investigate the influence of *smoking on the risk from radon*, dosimetric modelling was carried out which showed that smoking decreases radon dose to the lungs, whilst increasing it to other organs (Honorio da Silva et al. 2023). Speculation for the reason for this curious observation points to a thicker mucus lining and a slower clearance of mucus in the lungs of smokers, which results in a shielding effect of the early bronchial epithelial cells. Furthermore, through laboratory experiments, it could be seen that nicotine in cigarette smoke increases the DNA repair rate in bronchial epithelial cells following an alpha particle exposure, leading to more erroneous DNA repair, thereby increasing the risk of cancer formation (Boroumand et al. 2024). These results bring us closer to elucidating mechanisms for understanding the relationship between smoking and radon-induced lung cancer. This unexpected reduction in radon dose to epithelial cells in the smokers also has to be further investigated, more so in the context of new smoking habits such as vaping.

A survey of literature data concluded that there is only little evidence for *health effects of radon for diseases other than lung cancer* (Henyoh et al. 2024). Diseases that were considered in this study were circulatory disorders, cerebrovascular diseases, diabetes, digestive diseases, liver diseases and other forms of cancer. The paper highlights the need for better experimental and epidemiological strategies to ascertain any links between radon and these diseases. These findings are also supported by the analysis of German uranium miners cohort which did not provide convincing evidence for an association between radon exposure and death from diseases other than lung cancer (Fenske et al. 2025).

Work in RadoNorm is also significantly focussed on developing *adverse outcome pathways (AOPs) for various exposure situations*. Jaylet and colleagues (2022; 2023) have managed to develop a tool to determine an AOP for radiation-induced congenital microcephaly from existing literature data using a novel machine learning approach. To further characterise the risk in children, work is ongoing in the project to develop a biokinetic model for radon exposure in pregnant women and to ascertain the risk that radon in homes poses for cancer in children.

RadoNorm also tackles *risk from radon towards vulnerable populations* and lung dosimetry in children has shown that the smaller airway dimensions and thinner mucus layers lead to high absorbed doses in children compared to adults (Degenhardt et al. 2025). Moreover, the dose from the same radon concentration results in higher lung doses in people with asthma and chronic obstructive pulmonary disease (COPD) compared to healthy people (Honorio da Silva et al. 2024; Füri et al. 2025). These results pave the way for personalised dose assessment and protection strategies for vulnerable populations.

The Stochastic Lung Model (SLM) was also applied in RadoNorm to assess how anatomical and physiological variability influences lung dose following inhalation of radon progeny. Specifically, it was used to estimate lung doses in children, and in individuals with asthma and COPD, as well as to quantify the impact of smoking on respiratory deposition patterns. To enhance accessibility, the model is being implemented as a user-friendly web application, OpenSLM (https://slm.ek.hun-ren.hu/), which enables users to customise input parameters such as particle characteristics and breathing conditions. OpenSLM will provide downloadable data on regional and airway generation-specific deposition, supporting research and local exposure assessment. By adhering to FAIR (findable, accessible, interoperable, and re-usable) principles and combining computational precision with broad usability, the platform will contribute to advancing environmental health and inhalation dosimetry.

Complementing the FAIR-compliant development of OpenSLM, RadoNorm has also contributed to FAIR data management in the field of low-dose radiation research. Polgar et al. (2022) compiled a curated database of 101 in vitro clonogenic survival datasets from 46 published studies investigating low-dose hyper-radiosensitivity (HRS) and induced radioresistance (IRR). By digitising survival fraction data along with associated uncertainties and recording key experimental parameters the study addressed a critical gap in the accessibility of raw experimental data in this domain. The database openly available via the STORE^DB^ (Polgar et al. 2022), enables reproducible analyses, facilitates model development, and supports comparative studies.

A literature study investigating the *risk of cancer from ingesting naturally occurring radionuclides (NORs) through drinking water* highlighted a lack of reliable data to prove any risk and there is high uncertainty regarding the concentrations of radionuclides, leading one to conclude that there is little evidence of cancer risk from NORs in drinking water (Alimam and Auvinen 2025).

A newly developed toolkit was used to quantify *uncertainties in biokinetic model parameters for the calculation of dose coefficients* for defined scenarios of uranium-238 (U-238) ingestion by workers (Makumbi et al. 2025). The study showed that while the reference values of the International Commission on Radiological Protection (ICRP) are generally reliable, they may underestimate average doses and miss extreme cases. The sensitivity analysis identified the fraction of uranium absorbed from the alimentary tract into the blood as a key parameter that contributes to uncertainty hence emphasising the need for accurate estimation of this parameter. Similar concerns have been raised for inhalation exposures, where recent reviews highlight the role of parameter uncertainties in lung dose assessments, particularly in mining environments (Makumbi et al. 2024) While ICRP values are useful, the incorporation of additional statistical measures such as higher percentiles or the use of the mean as a reference value could improve radiation protection and dose assessment for U-238 ingestion.

Furthermore, biokinetic models were developed for *dose estimation for inhalation of radon and thoron progeny in underground mining environments*. The results suggest that miners performing Job Type 4 (dry drilling with poor ventilation) are at greater risk of lung cancer from radon progeny than those performing Job Type 1 (wet drilling with good ventilation), whereas the dose due to thoron progeny is higher for Job Type 1 than for Job Type 4 in spite of radon being the greater overall hazard (Makumbi and Spielmann 2024). Thoron exposure contributes significantly to lung dose variability, particularly in the bronchial and alveolar-interstitial regions, where miners are at increased risk of serious respiratory diseases such as lung cancer, COPD, and pulmonary fibrosis. From this study, recommendations were given calling for improved ventilation systems to reduce radon and thoron progeny concentrations, encourage use of advanced personal protective equipment for miners and health surveillance programmes for miners.

Notably, these efforts highlight the fruitful collaboration between dosimetrists and epidemiologists in the project, which is expected to result in more tangible output through the remaining deliverables and publications expected in the final months of the project.

**Fig. 1:**
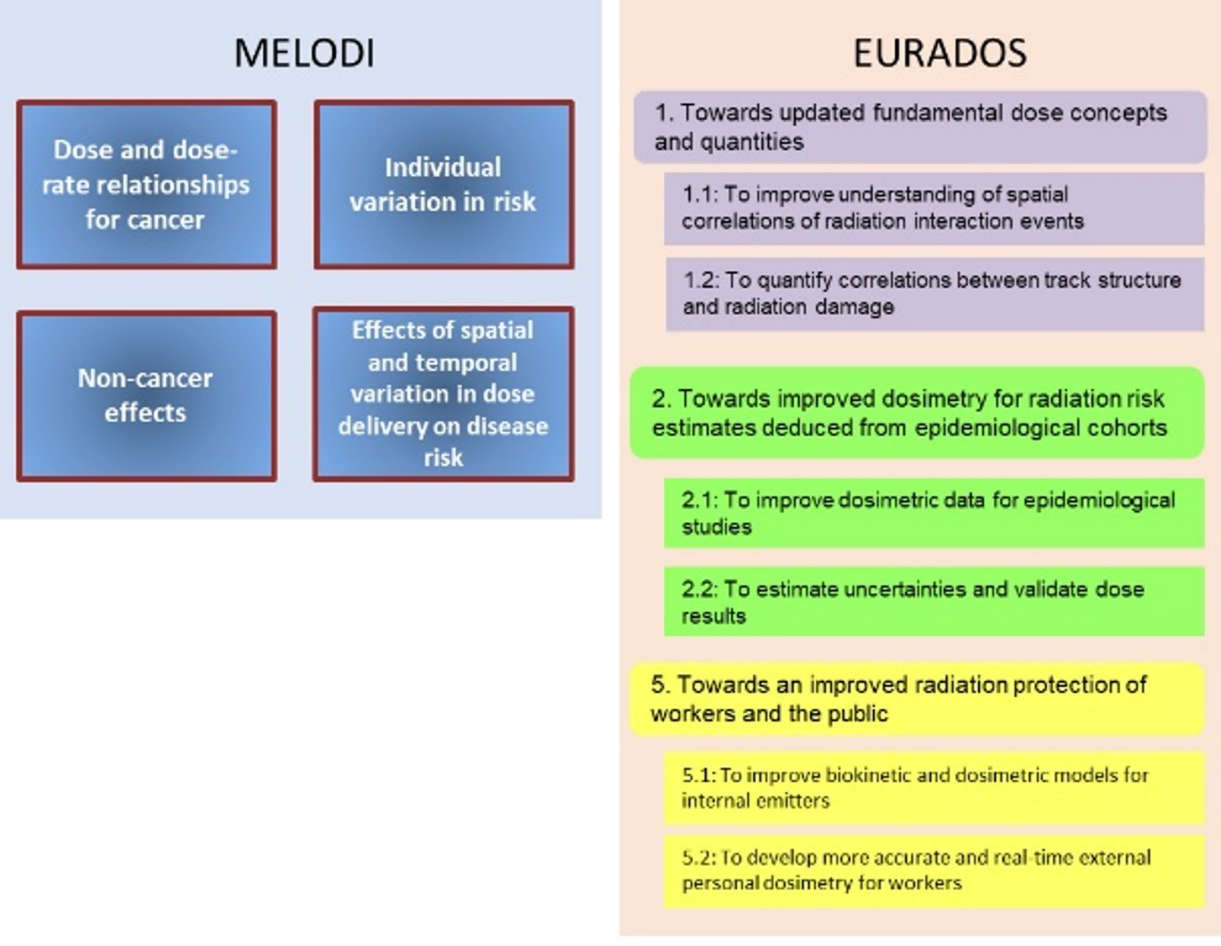
Contribution of RadoNorm results to SRAs of MELODI and EURADOS

The results contribute essentially to all four of MELODI’s topics: dose and dose-rate relationships for cancer, non-cancer effects, individual variation in risk, and effects of spatial and temporal variation in dose delivery on disease risk (Bouffler et al. 2022) as shown in Fig. 1. As such they also contribute to MELODI’s priorities outlined in Challenge A of the joint roadmap (Impens and Salomaa 2019). Some of EURADOS’ challenges from three of its visions are addressed (Harrison et al. 2021), for instance “To improve understanding of spatial correlations of radiation interaction events”, “To quantify correlations between track structure and radiation damage”, “To improve dosimetric data for epidemiological studies”, “To estimate uncertainties and validate dose results”, “To improve biokinetic and dosimetric models for internal emitters”.

### Exposure and mitigation

Several *intercomparison measurements for active radon monitors* were performed in RadoNorm. These comparisons encompassed budget radon monitors as well as research-grade measuring devices (Beck et al. 2024; Navrátilová Rovenská et al. 2024; Rey et al. 2024a, b). The results for the budget monitors showed discrepancies at lower radon levels, highlighted the influence of various parameters on their performance and shed light on issues of calibration. Taken together, the studies were able to put forward recommendations to the layperson for how to go about measuring with budget monitors, including placement, measurement period, evaluation and so forth (Feige et al. 2024), as well as give recommendations for the use of such monitors in underground workplaces. Concerning *occupational safety in underground workplaces* advances were made in developing software for calibrating devices in confined workplaces (Skubacz and Michalik 2022; Skubacz et al. 2024) and recommendations for conducting more accurate dose assessment in mines, considering measurement of potential alpha energy concentration (PAEC) (Grygier and Skubacz 2024; Vignaud et al. 2023). It is also important to determine the activity size distribution of radon progeny in such conditions and new technology has been developed which has so far been successful under laboratory conditions and is now ready for field testing (Fialova and Otahal 2024). Measurements of radon concentration and PAEC from active mines to tourist or legacy mines revealed high seasonal variability despite active ventilation protocols, revealing insights into the effects of mine drafts, along with high variability within the mines (Grygier et al. 2025).

For assessing *risk from indoor radon exposure*, models were developed to assess exposure to radon from anthropogenic origins such as radium-bearing waste and uranium mill tailings and showed the feasibility of using such calculation tools to aid expert risk assessment in buildings (Mansouri et al. 2023). Exposure to gamma radiation and radon exhalation from building materials was also assessed from a modelling perspective (Di Carlo et al. 2024) as well as with direct measurements (Maiorana 2025). While a variety of models exist to predict indoor radon concentration and gamma exposure from these materials, the choice of the model depends on its context of use. Moreover, direct measurements of radon exhalation, using the newly developed SIREN apparatus in RadoNorm, are much more accurate than models available to predict radon exhalation.

In terms of *mitigation strategies for reducing radon concentrations* inside buildings, assessment of the variability and sustainability of preventive and corrective actions for homes and workplaces showed that the durability and integrity of the barrier towards the ground is essential (Rudjord et al. 2024). A life-cycle assessment of various membranes available on the market revealed that high density polyethylene was seen to be the most promising membrane, offering optimum radon insulation, whilst having minimum environmental impact (Felicioni et al. 2023). Furthermore, failures associated with passive ventilation of the building are related to ingress of radon through openings and faults in this barrier. As such, when radon-proofing buildings, it is not just the membranes themselves that need to be considered but the way their joints are sealed and some techniques were found to be less effective than others (Jiránek et al. 2024). In this light, a prototype of an advanced measurement device for determining radon diffusion coefficients from these membranes was developed in the framework of the project (Jiránek and Froňka 2023). Radon concentrations may also rapidly increase following thermal retrofitting and changes of windows, especially for older houses, which shows that building energy efficiency policies also affect radon exposure indoors (Rudjord et al. 2024). Very high sustainability was seen for ventilation systems in studies from Czech Republic, Austria, and Norway, but compliance with technical procedures, operation and maintenance of the system is important. It is recommended, therefore, that radon preventive actions be verified by a long-term radon measurement after the building is occupied, and preferably be repeated periodically. In this context, it is important to mention a key factor influencing the indoor radon concentration that is ventilation. RadoNorm has contributed significantly to the objectification of indoor radon measurements by developing methods for determining the average air exchange rate using trace gases (Jílek and Froňka 2025). The report provides standardized methodological guidance for use of a passive integral tracer gas technique for determining average air exchange rate in a building or a workplace.

In the context of *outdoor radon exposure*, the movement of radon and its progeny in various environmental compartments was modelled (Hoftuft et al. 2024; Vives i Batlle 2025a). The model was able to shed light on several relevant mechanisms which are generally neglected in most assessment models. It is also able to assess the risk of uptake of radon progeny into plants and any consequent bioaccumulation, which could pose a risk to health. By coupling radon exhalation fluxes from the surface and meteorological data to a long-range atmospheric transport model, radon in the atmosphere was also modelled at a European scale and shows promise in complementing measurement networks and thereby providing estimates for population exposure levels (Hoftuft et al. 2024). Such efforts would significantly help improve maps delineating radon priority areas.

*NORs in the environment* pose risks to both humans and non-human biota and advances in radioecological studies have managed to clarify these risks. A model for radium migration in the soil shows the ability to predict sorption and desorption in various soil compartments based on the physicochemical properties of the soil (Serra-Ventura et al. 2024) and the parameters governing the mobility of uranium in a variety of contexts have been investigated (Vanhoudt et al. 2024; Vidal et al. 2024; Pelkonen et al. 2025), also including the influence of other biological entities. The use of soil-solution distribution coefficients (K_d_ values) for modelling combined with laboratory experiments have managed to convincingly elucidate sorption mechanisms and the main soil properties governing NORs in soils. Together with a model developed for assessing the combined effects of ionising radiation and chemical pollutants on wildlife (Vives i Batlle 2025b), new knowledge has been gained on risks to non-human biota, paving the way for better regulatory practice. Application of these results can be extended to NOR risk assessment in areas where mining is planned, such as the Fen complex in Norway. Moreover, these results show that symbiotic interactions between plants and microbes are able to influence the uptake of uranium into plants (Galeone et al. 2024) and that the microbial populations can be easily stimulated to reduce uranium(VI) to uranium(IV), rendering it non-bioavailable (Newman-Portela et al. 2024). Such insights have applications in the contexts of remediation strategies for sites contaminated by uranium and other NORs.

In terms of regulatory practice, there is a lack of guidance for certain scenarios that are not covered by the current guidelines (European Commission et al. 2003). One such instance is the use of sludge from groundwater treatment facilities that is reused as fertiliser in agriculture. For this scenario, screening levels in terms of activity concentrations of NORs fulfilling a given dose criteria were derived for this sludge (Venoso et al. 2024). The calculation tools and methods used in this assessment could potentially be applied to other scenarios in order to update the current guidelines.

For NORM-involving industries, registers were developed to help identify and assess risks associated to the presence of NORM in various industrial processes (Michalik et al. 2023; Mrdakovic Popic et al. 2023). These registers, which are freely available for download (RadoNorm 2022), can be applied to determine risks in any NORM-involving industry and can be easily used by both industry personnel and authorities to develop better governance policies for NORM-industries and processes to mitigate against high exposures to NORM.

**Fig. 2:**
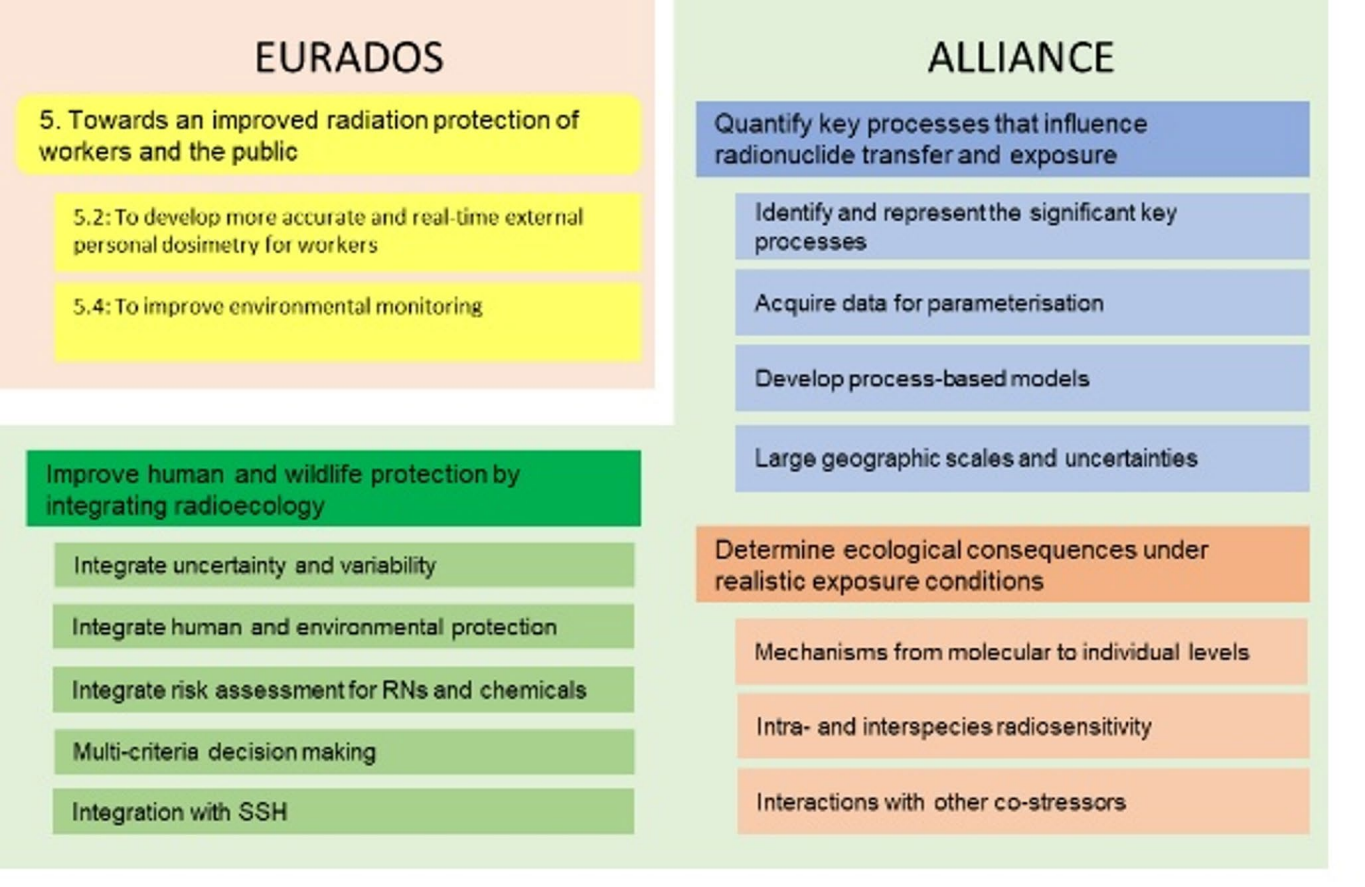
Contribution of RadoNorm results to SRAs of EURADOS and ALLIANCE

These results primarily address a plethora of research lines from the three challenges in the ALLIANCE platform (Gilbin et al. 2021): “Quantify key processes that influence radionuclide transfer and exposure”, “Determine ecological consequences under realistic exposure conditions” and “Improve human and wildlife protection by integrating radioecology” (Fig. 2). Overlap also exists with certain EURADOS challenges “Towards an improved radiation protection of workers and the public”.

Importantly, this work brought together engineers, physicists, modellers and architects to address cross-cutting issues in each of the above-mentioned topics.

### Risk communication and societal aspects

A number of institutions in RadoNorm conducted an ambitious pan-European survey in 16 countries to *measure risk perception*,* awareness and knowledge of radon*. The results were compiled and made publicly available for authorities and other stakeholders to gauge perception in various countries (RadoNorm 2024a). The results show in Slovenia that there is a weak association between the knowledge of radon and someone’s intention to take action to protect (testing and mitigating) (Perko et al. 2024). Determinants that strongly influenced people to take action were more to do with their understanding of the severity of consequences of radon exposure, their perceived susceptibility to risks, if radon is being tackled by family, friends and society, and what emotions are being processed through radon communication. This highlighted that effective communication interventions must go beyond merely raising awareness and knowledge.

In the context of communication, *framing* is also an important factor to consider. Also, among the Slovenian population, survey results showed that framing radon as an “indoor air pollutant” led to heightened risk perception than framing it as a “naturally occurring gas”, albeit, the opposite was true when it came down to intentions to test and mitigate against radon, i.e. framing as a “naturally occurring gas” led to higher intention to test and mitigate (Perko and Hevey 2024). In this light, a collection of social science methods such as surveys was made publicly available through the “social science toolbox of qualitative and quantitative methods” (RadoNorm 2023), together with training materials for authorities in particular to use these tools (Martell and Perko 2024).

Surveys and interviews conducted in Germany, Austria and Czech Republic also revealed a *dual radon reality* among the public, where radon is perceived as both beneficial and harmful (Himmelbauer et al. 2024). Naturally, the tendency of health bodies promoting the medicinal use of radon would frame radon as beneficial, focussing on its natural properties and luxurious experience among others. Communicating risks of radon therefore pose a problem to authorities who seek to protect people from high and prolonged exposures. Therefore, recommendations have been provided advising experts to be transparent and consider both realities in their communication campaigns, to provide context for each reality and to incorporate relevant stakeholders such as medical experts.

Furthermore, workshops among members of the public in Belgium and Slovenia showed that people require more government intervention in terms of establishing certification of mitigation companies, requiring radon safety certification such as in the case of building energy efficiency, greater advice and financial support from the state, and more interventions to raise awareness (Apers et al. 2023). The discussion highlighted the importance of holistic communication such as through doctors and architects and a variety of communication tools were suggested, which are currently under evaluation in RadoNorm research.

Since various efforts have previously been made in involving citizens in radiation research (Keller et al. 2019; Turcanu et al. 2020; Martell et al. 2023), RadoNorm aimed to create a sustainable *citizen science* network to raise awareness of radon and encourage testing and mitigation. For this, four pilot projects began in France, Hungary, Norway and Ireland (Martell et al. 2022). The projects themselves engaged a range of citizens and had slightly differing objectives. The lessons learned in these projects led to an open call for citizen science projects, spanning a maximum of six months with a maximum funding of 25,000 €. From this call, six projects were selected from the 19 proposals that were received. These six projects conducted in Spain, Slovenia, Slovakia, Poland and Italy were highly effective in raising radon awareness in their local regions and provided advice on how to go about testing for radon and mitigating it (Martell et al. 2025). They used various communication platforms such as local newspapers, radio stations, blogs, social media, etc., conducted tests in homes, workplaces and caves, and even spurred further actions from local authorities following the end of these projects (Source International 2025). Their level of success won them an honorary mention from 288 applications for the EU Prize for Citizen Science (ARS Electronica 2024). Consequently, these projects led to sustainable initiatives in their regions and their coordinators continue efforts ardently to make a compelling case for taking action against radon risks.

Furthermore, an investigation of the societal aspects of radiation protection related to *radon in geothermal energy installations* in Belgium and the Netherlands revealed a generally low risk perception among the public and industry personnel (Geysmans et al. 2024). This is mainly because of minimal risk resulting from exposure levels to ionising radiation in various processes being well-below the safety thresholds, as mentioned in interviews. The safety culture within the industry has a high standard due to continuous training and awareness raising. Moreover, it was mentioned that radiation protection should continue to be an aspect of the industry due to installations in new geological locations and the continuous amassing of NORM-containing by-products. Managing waste from these installations was highlighted as a concern and potential is being explored in how these residues can be reused. Such insights are important in the light of growing interest in geothermal energy as a means of clean and renewable energy.

It is also well-established that the construction industry continues to look for alternatives to clinker (being a large contributor to CO_2_ emissions) in cement for use as cementitious binders, which tend to contain substantial quantities of NORM (GCCA 2021). Interviews with representatives in the concrete industry revealed six overarching *factors that influence the use of NORM-containing products* such as cementitious binders: availability of by-products, financial factors, quality and performance, sustainability features, customer demand and acceptance of NORM-containing products (Love et al. 2023). Among end-users, the trends were similar, identifying issues pertaining to health, performance and economy as the driving factors, albeit varied in priority from country to country, looking at Belgian, Slovenian and Czech contexts (Love et al. 2025). The findings also showed that both industry and end-users look to governments and policymakers to devise better regulatory control to assure their safe and facilitated use in the market. Such insights are not only relevant to the construction industry and their aims to bring reduce carbon footprint, but also to a market with an ever-increasing assortment of NORM-containing products and processes.

**Fig. 3:**
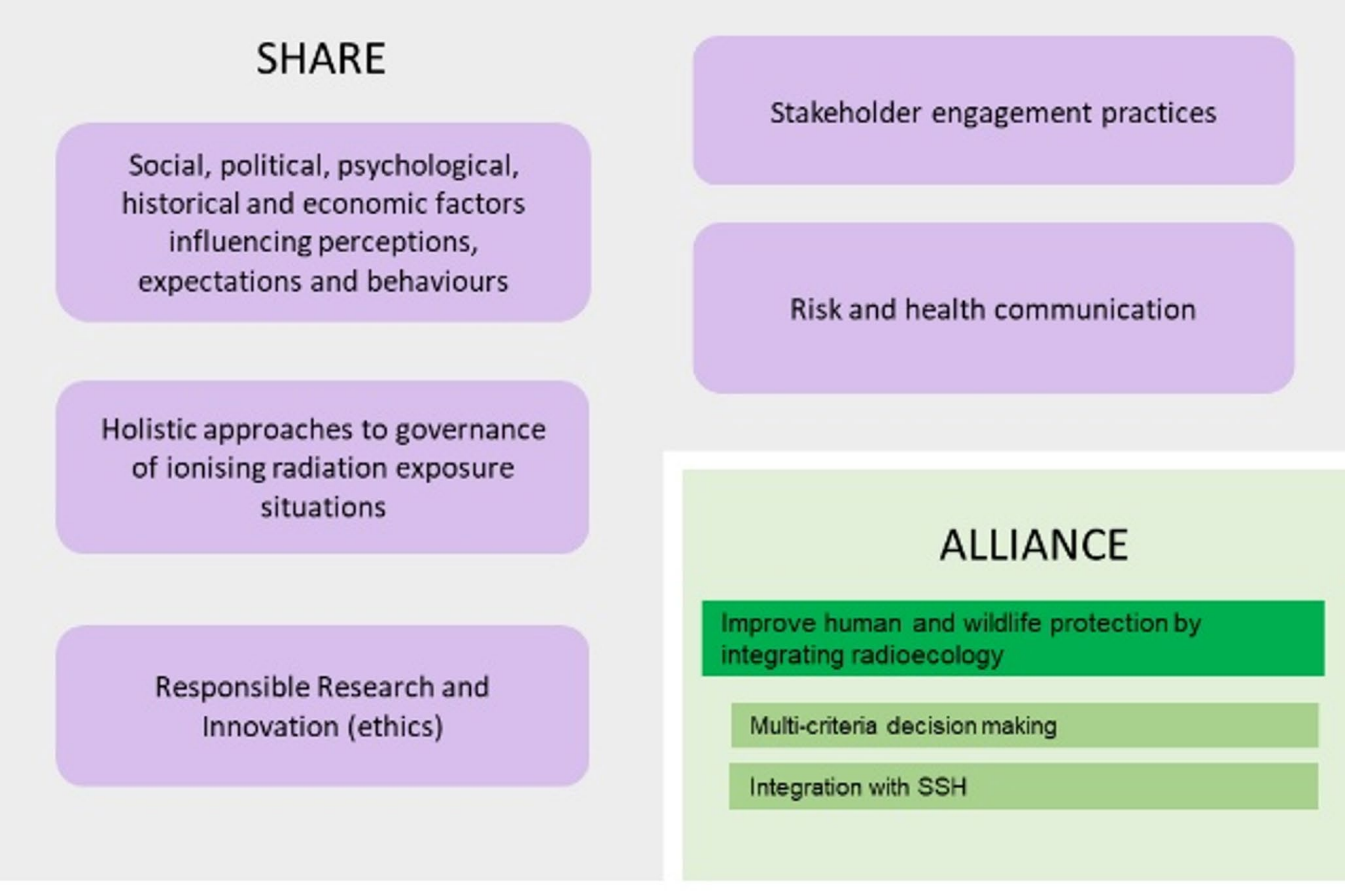
Contribution of RadoNorm results to SRAs of SHARE and ALLIANCE

Taken together, these results contribute primarily to the research lines of the SHARE platform to address (1) social, political, psychological, historical and economic factors influencing perceptions, expectations and behaviours, (2) holistic approaches to governance of ionising radiation exposure situations, (3) responsible Research and Innovation (ethics), (4) stakeholder engagement practices, and (5) risk and health communication (Perko et al. 2019; SHARE 2020) as shown in Fig. 3. Both SHARE and RadoNorm have benefitted immensely from an integration of the social sciences and humanities in radiation protection research, where notable collaborations include dialogue between social scientists and mitigation experts, authorities, and industry experts. The ALLIANCE platform has also profited from the integration of the social sciences and humanities topics, specifically in the case of NORM industries and processes.

### Education and training activities

In addition to the research itself, RadoNorm holistically incorporated the training of early career researchers (ECRs; including PhD students and early career postdoctoral fellows) and gave them a platform to encourage a bottom-up approach for sustainable competence (re)building in radiation protection research. Consequently, 25 PhD projects were funded, with the majority already having successfully received their doctorates. The total cohort of 36 ECRs organised themselves into the “Early Career Researcher Council”, where they organised regular meetings amongst themselves to discuss their projects, offer peer support, organise training courses and engage with senior personnel. This initiative helped in their networking and training, whilst also giving them the chance to further their knowledge in a multidisciplinary context. The travel grants offered within the framework of the project significantly spurred their efforts to come together, organise exchange visits, and disseminate RadoNorm output in conferences and other events.

Experience in the project has taught that funding alone is not enough to recruit and retain young researchers in radiation protection. Offering opportunities to take initiative, network and independently build something meaningful are effective means of keeping the motivation of ECRs high and encouraging them to pursue careers in radiation protection. The vastness of the RadoNorm consortium has also helped ECRs to find careers among partners, whilst also helping partners to collaborate more through ECRs participating in exchange visits and research stays.

An aspect that was not addressed in the project was mentoring, which has been an attractive initiative currently offered by other networks. Mentoring in this respect goes beyond the simple transfer of knowledge from PhD supervisor to candidate and should build a relationship that incorporates teaching, sponsoring, guidance, socialisation into a profession, and provision of counsel and moral support that allows the mentor to aid the mentee in the realisation of goals (Bussey-Jones et al. 2006). RadoNorm recommends that the radiation research platforms explore mentoring opportunities as a means of retaining young researchers and growing their interest in the field.

## Challenges for radon and NORM research

In many areas of risk management for radon and NORM, the groundwork has been substantially laid, which now needs to be picked up in terms of implementation into policy and practice. In this respect, further open questions and avenues of research were identified. This research is vital to continue effectively protecting EU citizens from risks from radon and NORM, whilst also contributing to the implementation of the European BSS, and incorporating other EU priorities such as sustainability, circular economy, beating cancer and climate change policies.

By integrating interdisciplinary evidence and addressing complexity, uncertainty, and societal values, this research will support the development of robust, evidence-based policies for radiation protection, ensuring effective management of radon and NORM exposures across Europe.

The consortium has formulated two overarching challenges: one for radon and one for NORM. The consortium appeals that these topics be incorporated into the SRAs of the platforms and into future funding schemes.

### Challenge 1: advancing radon research and bridging knowledge transfer to support effective implementation in radiation protection policy and practice

#### Scope

In RadoNorm, the quality assurance of measurement devices has been assessed, which has led to improvements in the measurement of indoor radon and assessment of exposure of workers. Procedures for quality assurance for these devices would now have to continue and also include thoron and progeny, which are known to contribute significantly to the total dose. The development of the thoron calibration lab at BfS during RadoNorm will help immensely in these efforts. This dose assessment will also have to be expanded to other criteria, previously not considered in risk assessments such as the effects of climate change.

RadoNorm managed to assess the available radon control technology and strategies to prevent and mitigate radon exposure and these would now have to be further refined since control technologies have barely changed in the past decades. Furthermore, methods to ensure safe and robust installation of both preventive and remedial measures should be developed so that the measures are cost-efficient and safe (avoid situations where the measure could lead to higher radon concentrations). Stakeholders will also need to come together to develop robust technologies and strategies to reduce indoor radon as practices have been seen to vary significantly across Europe. This includes more stringent policies governing building materials and their radon exhalation rates.

Moreover, development of biokinetic and biodosimetric models, together with their characterisation in terms of parameter uncertainties, for specific population groups and specific exposure conditions will have to continue, building upon knowledge gained in RadoNorm, and be expanded to ascertain the impact of assuming diverse parametrization of exposure scenarios. In RadoNorm, the effects of stressors such as smoking on radon dosimetry and biological effects were investigated but this research would now need to be expanded to the effects of modern trends in smoking (e.g. vaping) as well as multiple-hazard-exposure scenarios. In addition to lung cancer, genomic analysis conducted in RadoNorm revealed altered genes involved in cardiovascular, respiratory, neurocognitive and autoimmune diseases or syndromes, suggesting also non-cancer effects. These findings highlight the importance of risk communication in future campaigns, given that lung cancer is the primary risk being communicated in campaigns today.

Finally, the knowledge gained through RadoNorm now has to be implemented into policy and practice. This should lead to the development of tailored policies in European countries for implementing their radon action plans, since a harmonisation of procedures has proved difficult.

#### Breakthrough research topics

Topics in this area that would achieve profound impact would be:

- developing benchmarking standards for manufacturers of radon monitors, better calibration procedures, including the development of radon progeny primary standards, and undertaking more robust testing.
- developing new innovative measurement systems and principals for measuring thoron and its progeny.
- developing methods to measure the radon progeny attached fraction in workplaces where models fail to predict it.
- redefining the national mapping of radon-prone areas on the basis of national databases of radon levels inside buildings and the geological radon risk map.
- widening the scope of national surveys with consideration of thoron/thoron decay products exposure.
- describing and understanding the prolonged effects of thoron on tissues after clearance from the lungs and studying the vulnerability of tissues such as the lungs, bronchi and kidneys.
- using biokinetic and dosimetric models and their uncertainty bands to better design workplace-specific protective measures against thoron.
- considering the effects of external factors (e.g. energy savings, climate change, public policy and use of personal protection) on exposure levels.
- laying down standardised requirements for radon barriers for building mitigation.
- evaluating the contribution of radon exhalation rates from buildings to total radon dose (from lab measurements to real life).
- involving building professionals and other relevant stakeholders like ISO committees to improve radon control strategies and their safe implementation, whilst developing training and tools for these professionals.
- developing reference parameter distributions for all relevant input and model parameters for biokinetic and dosimetric models used in dose assessment for workers and populations exposed to radon.
- developing a mechanistic understanding of the behaviour of radon and its progenies in the human body to refine the existing compartment models and base them better on chemical and physiological processes tailoring treatments to specific molecular and biological profiles.
- investigating coeffects of radon and new tobacco products and smoking practices (vaping, etc.) on cancer and non-cancer effects.
- investigating cancer effects (including those with notable findings from previous studies, such as lymphomas, stomach cancer, liver cancer, skin cancer, and breast cancer) and non-cancer effects (particularly circulatory system related) of radon, including clinical implications for precision medicine and the potential development of targeted therapies.
- investigating radon-related mechanisms involving other pathways, such as the immune system, metabolism, epigenetics, and alternative processes and the subsequent development of adverse outcome pathways (AOP).
- modelling the effects of radon at the macroscopic level on wildlife populations, and performing field studies to validate such models.
- coordinating efforts to synchronise radon interventions with broader initiatives such as indoor air quality campaigns, “EU Beating Cancer plan,” energy efficiency programs, the EU Bauhaus initiative, and other related EU efforts through engagement of authorities and policy makers.
- conducting ethics research to evaluate the impact of policies and to optimise data availability to support the EU open science policy and general data protection regulation.
- integrating the exposure to radon and co-existing exposure to other NOR in workplaces in one coherent monitoring and control system.

#### Impact

Research tackling the topics listed above would help to provide a more holistic assessment of radon dose to individuals and populations, with an integration of human and wildlife assessment. It would help policy makers to develop trust in the devices they recommend to the public to increase radon testing rates. Moreover, the already fruitful collaboration between institutes involved in metrology can be sustained to build on intercomparison exercises and robustly evaluate new devices coming into the market, as the societal issue of radon begins to grow. Radon mitigation strategies would be not just improved but finally implemented to reduce indoor radon concentrations. Tailored approaches to protect specific population groups more vulnerable to radon can be developed, including better occupational protection for those exposed to significantly high radon levels (e.g. miners). Policymakers will also be able to better implement risk communication strategies through their radon action plans and have a more comprehensive understanding of the health risks involved. Ultimately, it would help EU members states to implement the European BSS to limit exposure of the population to radon and contain exposure within the given dose restrictions.

### Challenge 2: radiation protection in sustainable management of NORM

#### Scope

Management of naturally occurring radionuclides has to address many open issues such as governance and regulation for NORM-involving industries, management of NORM-residues and waste resulting from industrial applications, management and remediation of legacy sites, as well as assessing the transfer, migration behaviour and effects of radionuclides in the environment.

Within RadoNorm, tools for identifying and classifying NORM-industries were developed, that now need to be implemented in practice. In the context of circular economy and environmental policies, a clearer classification of NORM-residues stemming from industrial processes is needed as either reusable by-products or unusable NORM-waste. The use of NORM-containing by-products is already underway in certain industries, such as building materials. However, the governance of these products to avoid mismanagement and unintended exposure, such as in the former cases of their mismanaged reuse in building materials in some EU countries, is still lacking. In RadoNorm, various scenarios were assessed to provide clearance levels for industries where NORM is reused, such as in the fertiliser industry, and these scenarios will have to be expanded in order to provide evidence-based policy advice towards an EU-wide harmonisation.

This will also require socio-psychological research at both individual and societal levels on the use of NORM products and processes, taking cultural differences into account, as identified within RadoNorm. Consequently, a diverse range of stakeholders—including authorities, legacy site operators, and industry representatives—must be engaged in discussions to improve and harmonise NORM governance across Europe, especially in light of new technologies, for instance geothermal energy production, new building materials, remediation techniques, etc. These efforts should focus on developing a sustainable framework that promotes best practices and capitalises on opportunities for circular economy.

#### Breakthrough research topics

Topics in this area that would achieve profound impact would be:

- developing strategies to deal with liquid NORM in various exposure situations and different ecosystems.
- establishing science-based guidance and clearance levels (accounting for all exposure pathways) for NORM in different scenarios not covered by current guidance (building materials and common goods incorporating NORM, etc.) considering also Life Cycle Assessment (LCA).
- exploring further possibilities for NORM reuse, in line with circular economy, zero waste & green deal, UN sustainable goals, etc.
- integrating NORM-related hazard management with existing system of non-radioactive waste and occupational health and safety management.
- establishing guidance for NORM sampling, characterisation and especially NORM reference material that are currently missing.
- further validation of models for the environmental transport and transfer of NORM (including radon) in well-characterised scenarios, developing them into tools that can be used for impact assessment and the assessment of remediation options of legacy sites.
- involving stakeholders and owners of NORM sites to support accessibility for field work and verification of currently used or newly developed radioecological models.
- investigating risk communication of health risks from NORM among stakeholders.
- investigating social, environmental and economic sustainability of NORM-containing products or industries (i.e. their acceptance in society).
- developing and testing sustainable and cost-effective bioremediation strategies for soil and water beyond cost- and energy-intensive methods and transfer these to industrial applications.
- identifying indicators and conducting multi-criteria decision analysis (MCDA) for managing risks at legacy sites.
- exploring remediation and mining strategies for rare earth elements at NORM-affected legacy sites.
- improving upon and testing out radioecology models and expanding them to assess the effects of mixed contaminants on non-human biota (including toxicodynamic-toxicokinetic modelling, population modelling, adverse outcome pathways (AOP) and possibly using artificial intelligence).
- developing robust and cost-effective methods to better monitor the ecological status of sites contaminated with elevated levels of NORM to establish a baseline of present environmental conditions and screen remediation success.
- understanding how environmental stressors influence the uptake and biodistribution of NORM in epidemiological studies and in non-human biota (including impacts on dosimetry and long-term effects) improving risk assessment processes in health and ecological contexts.
- conducting ethical research to evaluate the impact of policies and to optimise data availability to support the EU open science policy and general data protection regulation.

#### Impact

Progress in these areas will bring legislation closer to a harmonisation of policies across Europe, which is needed, especially when governing the use of NORM-containing materials. Exploring alternative materials can help in reducing CO_2_ footprints and encourage circular economy, avoiding environmental pollution resulting from dumping of toxic, contaminant waste. Further investigating remediation strategies could help to develop sustainable and cost-efficient remediation processes which are currently chemical- and energy-intensive and could include strategies for the extraction of essential isotopes for use in medical applications or rare earth elements for new technology from legacy mining sites. Moreover, these would lead to a holistic understanding of risks for NORM-industry workers, end-users of NORM-containing products and populations living close to NORM industry, taking into consideration both human and environmental risks and help to assess risks better through the lens of a One Health approach and support sustainable development goals (SDGs) of the United Nations.

## Discussion with the radiation research platforms and other stakeholders

WP8 of RadoNorm is dedicated to communication, dissemination and exploitation of results. As part of these activities, open events are organised to present and discuss RadoNorm achievements with the international community. So far, this has taken place through monthly online webinars, as part of the RadoNorm Research Seminar series (RadoNorm 2024b), as well as through the project’s annual meetings, where external participants from renowned radiation protection institutions are invited to actively participate in panel discussions. The hybrid nature of the past few annual meetings has allowed extensive participation and exchange of ideas regarding the scientific outcomes of RadoNorm. More information regarding the annual meetings can be found in the published minutes (Birschwilks et al. 2021; John et al. 2022, 2023, 2024).

Most notable among the events organised, however, was the recent RadoNorm Showcase Meeting, organised on 27th March 2025 at the Institute of Natural Sciences in Brussels. The event focussed on: (1) presenting RadoNorm achievements and contributions to radiation risk management and building competence in the field; (2) highlighting areas that require further research to protect people and the environment from the effects of radon and NORM ionising radiation; and (3) fostering dialogue on future pathways for innovation and collaboration. This full-day event presented a great majority of the results highlighted in Overview of achievements and recommendations and contributions to European Radiation Research Platforms, followed by panel discussion to examine the impact of the results on representing organisations and their relevance for the EU Basic Safety Standards. Representing organisations were therefore asked to share their positions on implementation of these results and what future research is now needed. Three discussions around the topics of “health effects and risks”, “exposure and mitigation”, and “risk communication and social aspects” took place where representatives could voice their opinions.

This section will present feedback from the representing organisations at each of the three panel discussions on the results of RadoNorm. The organisations were invited to present their positions to the specified topics as shown in Table 1.

**Table 1:** Contributions from international organisations to the themes of the radonorm showcase meeting

Health effects and risks	Exposure and mitigation	Risk communication and social aspects
*MELODI*	*European Commission * *DG ENER*	*SHARE*
*EURADOS*	*ALLIANCE*	*Authorities (SNSA)*
*IARC/WHO*	*HERCA*	*HERCA*
*PIANOFORTE*	*PIANOFORTE*	*PIANOFORTE*
	*Authorities (ISS)*	*Authorities (BMUV*^1^)
	*Industry (RadoNova)*	

^1^The organisational decree issued by the Federal Chancellor of Germany on 6 May 2025 changed the structure and name of the ministry to BMUKN: Bundesministerium für Umwelt, Klimaschutz, Naturschutz und nukleare Sicherheit/Federal Ministry for the Environment, Climate Action, Nature Conservation and Nuclear Safety

### Health effects and risks

This panel discussion featured representation from Multidisciplinary European Low Dose Initiative (MELODI) by Fieke Dekkers, European Radiation Dosimetry Group (EURADOS) by Arturo Vargas and Balázs Madas, International Agency for Research on Cancer (IARC/WHO) by Evgenia Ostroumova and the European Partnership for Radiation Protection Research (PIANOFORTE) by Jacqueline Garnier-Laplace. The following is a summary of the discussion and the statements put forward.

The width of RadoNorm is essential as it incorporates every aspect of radiation protection together: RadoNorm brought together scientists from very different disciplines: from health effects, dosimetry, societal aspects, mitigation, etc. The inclusion of thoron in the project was an important part because it influences especially the evaluation of low dose exposure of radiation. Other noteworthy results are related to internal exposures and individual differences in received doses such as with co-exposures with smoking and other chemicals, and for specific subpopulations such as children and those suffering from lung diseases.

RadoNorm results show that models for thoron assessment can be applied from radon models but not directly. The results pertaining to a fingerprint for radon-induced lung cancer might be useful in epidemiological studies by improving the understanding of different exposures and their specific effects. RadoNorm has provided information on the radiation induced effects other than lung cancer, even though the data is not enough to provide a definitive answer yet. Results contribute to shape of the dose-response curve for cancer and non-cancer, which is part of the MELODI SRA. The low dose effect is especially essential for evaluation of radon in understanding the effects of radiation in general. As such, the data can be used to evaluate the low dose exposure effects more effectively.

Relevant RadoNorm results pertain to the application of radon dosimetric models and the development of metrology since no comprehensive metrology system is as yet available. Consequently, the traceability of the measurement of the unattached fraction of radon is still lacking, and monitors for measuring unattached fraction in underground workplaces is needed, for which RadoNorm results make progress.

The research about the radon attached and unattached fractions is important, as these fractions might differ substantially in different scenarios. Smoking risk on radon exposure has to be further investigated, for instance, in differentiating between smoking and radon-induced lung cancers/deaths in multiple exposure scenarios.

Something to consider in the future would be addressing how new policies for building energy efficiency affect health through ionising radiation exposure.

Outcomes of RadoNorm have full relevance for policy making. The IARC working groups on carcinogenic evaluation will use the data provided by RadoNorm in the evaluation of risks. The more refined evaluation of doses provided in RadoNorm will help lead to a future with less cancers. The results of RadoNorm can be used to help also other parts of the world such as Asia or South America. RadoNorm provides further information for the WHO database on radon. Improvements in dosimetry and modelling provide the best available data for health risk assessment.

Radon exposure and the risk of cancers in children was the most important research question that should be now further explored to ascertain risk. The continuation of the research on the combination exposures of radiation and other agents such as smoking or chemicals would be prudent.

PIANOFORTE acknowledges the scientific successes of RadoNorm and the attempts to bridge the full way from research to society, and from one large topic to another. PIANOFORTE is structured to identify research needs for future open calls by stakeholder involvement. It could be useful to combine the results of RadoNorm with broader radiation protection topics through appropriate PIANOFORTE open calls (such as Topic 2 of the third call in 2025: Developing an integrated approach for risk assessment and evaluation from environmental exposure to ionising radiation). PIANOFORTE positions itself as a platform for dialogue on these and other emerging research needs, provided these are supported by a broad and relevant stakeholder base, from practice to academia.

### Exposure and mitigation

This panel discussion featured representation from European Commission DG ENER by Giorgia Cinelli, the European Radioecological Alliance by Rodolphe Gilbin, Heads of the Radiation Protection Competent Authorities (HERCA) by Boris Dehandschutter, PIANOFORTE by Florian Rauser, Istituto Superiore di Sanità (ISS) by Christian Di Carlo and RadoNova by José - Luis Gutiérrez Villanova. The following is a summary of the discussion and the statements put forward.

Important results pertain to the registers provided for NORM sites and simplification of radioecological models for optimised use. However, this simplification should rely on science. Therefore, it is still interesting to know the specific behaviour of NOR in soils and waters, and to consider their speciation. Moreover, the protection of non-human biota and its inclusion in a holistic assessment for both human and non-human biota is important along with robust evaluation of mitigation systems. Multi-contaminant exposures still have to be addressed. These efforts directly support the implementation of European Basic Safety Standards and broader EU health and environmental policies.

In addition, the modelling of combined effects of ionising radiation and chemical pollutants on wildlife populations should be further developed. Mechanistic approaches such as adverse outcome pathways (AOPs) and toxicokinetic-toxicodynamic (TKTD) models may help strengthen ecological risk assessment.

In terms of the Green Deal and in light of circular economy issues, research has to ascertain how NORM exposure compares to other exposures. Moreover, the effect of human protection measures on the environment should be studied — this includes assessing trade-offs between energy efficiency (e.g. ventilation) and radon mitigation. Finally, new technologies should now be used to update radon maps, integrating artificial intelligence, geological data, and real-time sensor networks to better target mitigation actions.

Recommendations provided by the scientific community, such as RadoNorm, represent a practical and useful support for the national competent authorities in implementing their Radon Action Plans and meeting the requirements for radon in workplaces. These include recommendations for radon measurements in underground workplaces and the important results regarding the public awareness raising.

Other important results relate to the environmental impacts of different material types of radon-proof membranes and the results on the “good balance” between energy savings and indoor radon mitigation in buildings. Research should focus on generating results that can support authorities to implement the directive. Moreover, a clear characterisation of NORM sites is still needed, along with a clear assessment of workplaces with enhanced radon levels.

While radon research has already reached the level of implementation, it has to be acknowledged that NORM research is still in its early stages. Fundamental knowledge on NORM risks and mitigation strategies needs therefore still to be included in future research.

RadoNorm results on measurements and underground workplaces are relevant and can have practical implementation. Regulation of NORM legacy sites has to move forward to incorporate aspects of mixed contaminants. More tools are needed to perform the risk assessment at these sites. Linking to circular economy, the integration of NORM in building materials needs to go beyond the scientific issues it raises and account for the perception of the society towards the use of such materials. Radon mitigation in terms of energy consumption strategies and chemical interaction between radon-proofing techniques should be studied.

The integration and interdisciplinarity of the work in RadoNorm were noteworthy. The research even brought together both radon and NORM in certain research topics such as in the case of construction materials. Future research should discuss how to go about radon mitigation in cases where it is difficult or not possible, e.g. historical buildings, and how to improve occupational safety in such cases. Personal radon exposure in buildings with high indoor radon concentrations where remediation is not an option should be further investigated. Additionally, societal aspects should be further considered in detail.

RadoNorm was the first European project to specifically address the contribution of building materials to indoor radon concentrations. This issue was tackled from two complementary perspectives: modelling and direct measurement of indoor radon levels.

A comprehensive review of existing models for simulating indoor radon accumulation was conducted. The review critically assessed each model, clearly highlighting their strengths and limitations. In addition, a significant enhancement to the most flexible and comprehensive existing model was developed as part of the project.

An innovative apparatus was tested to systematically measure radon exhalation directly from existing constructions. This novel device was used to assess radon exhalation from walls made of building materials that had previously been characterised using sample-based measurements. These activities represent the first attempt to establish an experimental correlation between measurements taken from small samples—currently the most common and almost exclusive method—and in situ measurements conducted directly on walls. The latter is crucial for accounting for the influence of construction techniques and environmental conditions on actual radon exhalation rates. Identifying this correlation, or demonstrating its absence, could foster a more informed use of sample-based measurements in evaluating the contribution of building materials to indoor radon levels.

The research advancements achieved through the RadoNorm project define a framework in which:

- the generation and transport of radon through soil and building materials,
- the accumulation of indoor radon concentrations from various sources,
- the ingrowth of radon progeny, their attachment to aerosols, and adsorption on indoor surfaces, and.
- the equivalent tissue doses and effective dose resulting from specific granulometric distributions of radon progeny.

are well understood and can be accurately simulated. A major challenge for the near future is the development of a comprehensive model capable of assessing radon-related health risks starting from site-specific radon generation processes.

### Risk communication and societal aspects

This panel discussion featured representation from the Social Sciences and Research in Ionising Radiation Research platform (SHARE) by Yevgeniya Tomkiv, Slovenian Nuclear Safety Administration (SNSA) by Helena Janžekovič, HERCA by Bernd Hoffmann, PIANOFORTE by Marie Davídková and the Federal Ministry for the Environment, Nature Conservation, Nuclear Safety and Consumer Protection (BMUV) by Kaitlin Kammerlander. The following is a summary of the discussion and the statements put forward.

The RadoNorm website is a source of useful information. However, RadoNorm results have to be available also after the end of the project, namely, in decades to come. Ideally the information should be made more accessible and user friendly.

Effective communication of uncertainties regarding dose assessment to the general public should be investigated. Regulators are also challenged to go ahead with a control over exposures related to natural radiation due to the lack of available data from NORM industries as presented in RadoNorm results.

Future research should investigate the use of FFP2 masks as a protection measure against radon, radon risks affected by air pollution and influence of environmental changes on exposures related to natural radiation.

The lack of social scientists in radiation protection has become evident and we need to motivate young scientists including social scientists in particular to join the field of radiation protection research. In terms of application and implementation, we need to identify the correct stakeholders involved. Do we have the same stakeholder groups that deal with both radon and NORM, and where are the differences in communication strategies?

The citizen science projects were able to effectively engage citizens. The outcomes showed that citizen science is useful to raise awareness, especially in the field of radon. But at the same time, the core of each citizen science project should be a scientific question. To find new and adequate scientific questions for such projects is difficult. Such projects, which may also become self-sustaining, require sufficient resources and time.

The communication effect of citizen science projects in RadoNorm highlighted that the response of citizens to mitigation strategies was positive. Communication with the public during any process of risk mitigation is important. RadoNorm research on communication also shows there is no unified approach to communication across Europe and therefore points to the development of tailored communication strategies.

Inclusion of social sciences in this broad and self-evident manner is appreciated in RadoNorm research. The progress has shown that social science pertaining to radon is still far ahead of social sciences in the indoor air quality field.

The RadoNorm community incorporating multiple diverse actors could be kept alive by the involved actors, e.g., using the annual European Radiation Protection Week conference as a platform. The RadoNorm community should also integrate into longstanding, existing communities, such as the one on “indoor air quality” to integrate Radon related recommendations deeper into regulatory practice.

## Summary and outlook

The success of RadoNorm over its 5-year span in achieving its objectives and deliverables has led to considerable progress in assessing and managing risks from radon and NORM. The response from the international community to this progress has resonated well with the project’s original goals for the exploitation of its results and has proven the commitment in RadoNorm to making its research as relevant as possible. While RadoNorm expects much more of its results to be published towards the very end of the project (31 August 2025) and the months thereafter, its contributions to implementation of the European BSS, beating cancer, improving indoor air quality, improving circular economy and other European policies are staggering.

Following an evaluation of its progress, the consortium identified priorities for future research that should be taken up by radiation protection research for radon and NORM. Interestingly, this opinion was also echoed by several stakeholders at the RadoNorm Showcase Meeting, especially pertaining to a greater fundamental understanding of health risks, better occupational protection, better integration of social sciences and a more governed use of NORM-containing products in light of circular economy.

As RadoNorm has now established a solid basis for radon and NORM research, it is vital that the radiation research platforms continue the momentum gained and encourage funding of research referring to these topics, especially with the dawn of novel technologies such as AI, which could vastly improve risk assessment. Advances in RadoNorm have shed light on pressing issues that need to be addressed, not simply from a radiation protection standpoint but also related to global health and environmental issues. A greater urge to continue this research goes out to PIANOFORTE as the sole European partnership in radiation protection. Importantly, this would be in line with PIANOFORTE’s goals to enhance public and occupational health and protection of the environment, as well as to support its alignment to the agenda of the European Commission (2024–2029) by fostering quality of life, competitiveness, a risk-informed society and a choice of energy sources for climate change mitigation (Garnier-Laplace et al. 2025). As stipulated by Garnier-Laplace and colleagues (2025), RadoNorm too firmly advocates for consistent allocation of European funding for radiation protection research in the upcoming EU Framework Programme 10 in Euratom to not only meet these research needs but to ensure radiation protection is fit-for-purpose and encourage sustainable progress.

Furthermore, the added value and practicality of large projects such as RadoNorm should not be overlooked. The level of interdisciplinary work enabled breakthroughs in research on a scale never seen before and the collaboration in this regard was acknowledged and admired by various stakeholders. Collaboration through this large-scale project more notably took advantage of the combined expertise among a number of European institutions and consequently strengthened the fruitful partnerships among them.

## Supplementary Information

Below is the link to the electronic supplementary material.

Supplementary Material 1

Supplementary Material 2

Supplementary Material 3

## Data Availability

No datasets were generated or analysed during the current study.
