# Supplementary figures and images for: Contributions of the radonorm project to European and international radiation protection research
Source: Radiat Environ Biophys. 2025 Oct 18;64(4):561–79. doi: 10.1007/s00411-025-01156-w (PMC12701043; doi:10.1007/s00411-025-01156-w)

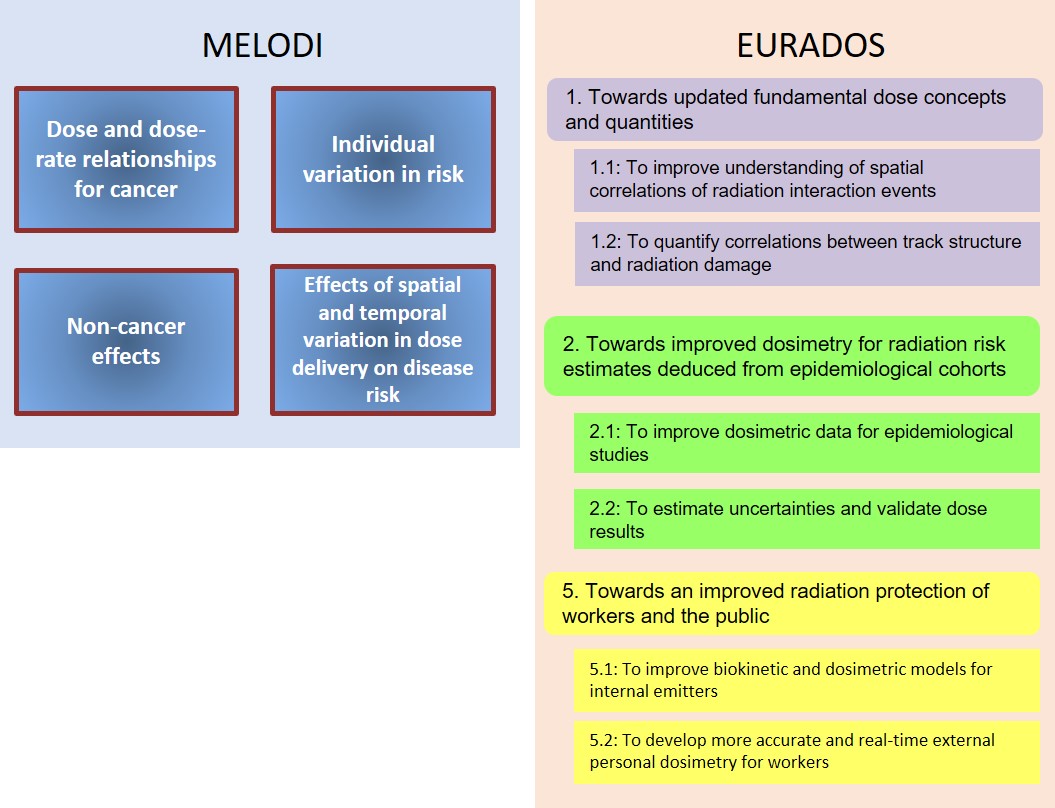

Supplement: Supplementary file 1 — Supplementary Material 1 [file 411_2025_1156_MOESM1_ESM.jpg]

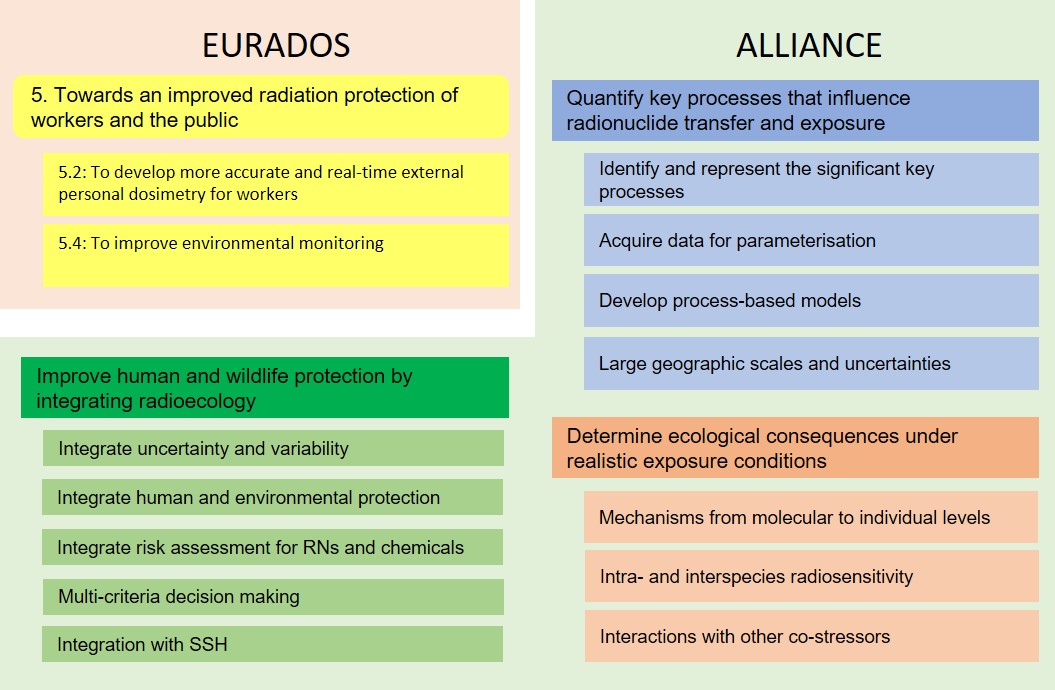

Supplement: Supplementary file 2 — Supplementary Material 2 [file 411_2025_1156_MOESM2_ESM.jpg]

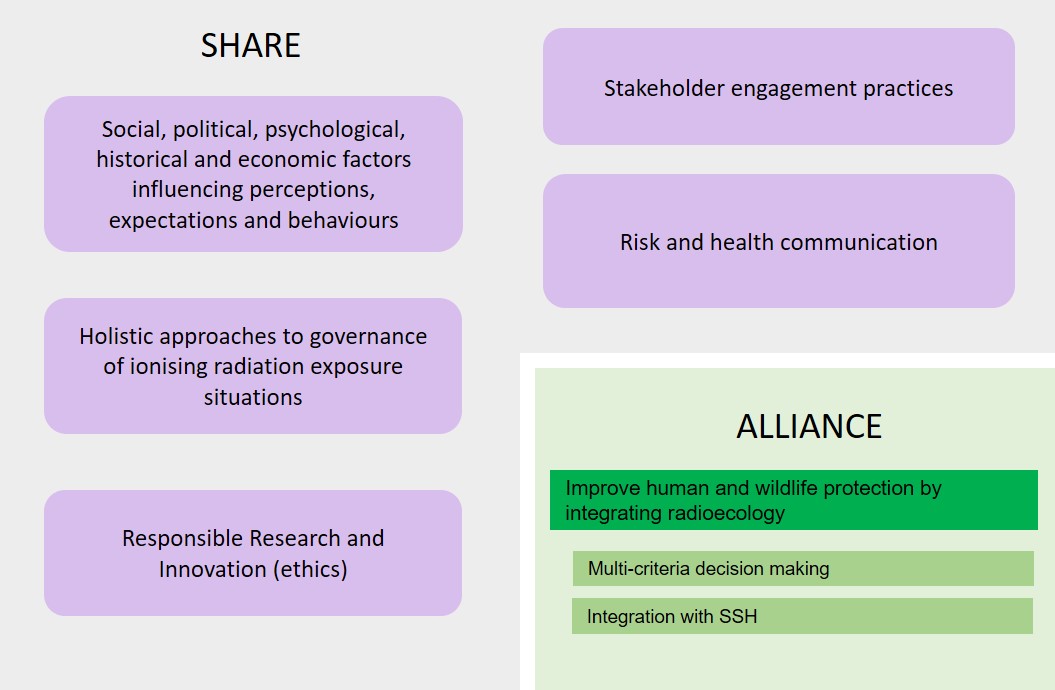

Supplement: Supplementary file 3 — Supplementary Material 3 [file 411_2025_1156_MOESM3_ESM.jpg]
